# Temperament, Character, and Depressive Symptoms during Pregnancy: A Study of a Japanese Population

**DOI:** 10.1155/2013/140169

**Published:** 2013-12-22

**Authors:** Mariko Minatani, Sachiko Kita, Yukiko Ohashi, Toshinori Kitamura, Megumi Haruna, Kyoko Sakanashi, Tomoko Tanaka

**Affiliations:** ^1^Division of Health Sciences & Nursing, Department of Midwifery and Women's Health, Graduate School of Medicine, University of Tokyo, 7-3-1 Hongo, Bunkyo, Tokyo 113-0033, Japan; ^2^Kitamura Institute of Mental Health Tokyo, 101 Akasaka 8-5-13, Minato, Tokyo 107-0052, Japan; ^3^Nagoya University, 65 Tsurumai-cho, Showa, Nagoya, Aichi 466-8550, Japan; ^4^Department of Women's Health and Mother-Child Nursing, Faculty of Life Sciences, Kumamoto University, 4-24-1 Kuhonji, Chuo, Kumamoto 862-0976, Japan; ^5^Aso Health Center, 1204 Uchimaki, Aso, Kumamoto 869-2301, Japan

## Abstract

*Background*. To examine the effects of temperament and character domains on depression during pregnancy. *Methods*. We examined 601 pregnant women using a questionnaire that included the Edinburgh Postnatal Depression Scale (EPDS), the Temperament and Character Inventory (TCI), and demographic variables. *Results*. In a hierarchical regression analysis, severity of depression during pregnancy was predicted by the women's negative response towards the current pregnancy, low self-directedness, and high harm avoidance, persistence, and self-transcendence. *Conclusion*. Depression during pregnancy is predicted by personality traits as well as women's negative attitudes towards the current pregnancy.

## 1. Introduction

The World Mental Health Survey [1], conducted in 17 countries, found that on average about one in 20 people reported having had an episode of depression. The prevalence of depression among women is two to three times higher than that among men. Depression is one of the most serious health problems among women [2], with younger women affected more frequently than middle-age women [3].

Personality is one of the major factors associated with depression [4–7]. Personality traits predict the course and treatment response of depression [8]. Cloninger et al. [9] proposed a psychobiological model that included both temperament and character domains. According to their theory, the temperament domain consists of four dimensions: harm avoidance (HA), novelty seeking (NS), reward dependence (RD), and persistence (PS). The character domain consists of three dimensions: self-directedness (SD), cooperativeness (CO), and self-transcendence (ST). As a measure of these dimensions, they developed the Temperament and Character Inventory (TCI) [10].

Many studies have used the TCI to explore the relationship between depression on the one hand and temperament and character dimensions on the other. Most of these studies demonstrated that individuals with depressive disorder or depression were more likely to have high HA and low SD and CO [6, 7, 11–16]. No consensus, however, has been reached regarding the relationship between PS and depression. While some researchers [12, 17–19] have reported *high* PS among those with depression, others have found *low* PS among those with depression [7, 20, 21], depressive disorder [22, 23], and bipolar disorder [22, 24–26]. It is of interest to us that PS was reported to be low among individuals with postpartum depression [27].

Depression that appears during pregnancy is called antenatal depression. The prevalence of this condition has been reported to be between 4% and 29% [28, 29], and it has been associated with unwanted and unplanned pregnancy, high neuroticism, attitudes towards the present pregnancy, lack of social support, and pregnancy complications [28, 30, 31].

Depression and depressive symptoms during pregnancy impact several perinatal health outcomes, including preterm delivery [32], low birth weight, intrauterine growth restriction [33], diabetes [34], and postpartum depression [35]. It is therefore of clinical as well as research importance to identify pregnant women with depression.

However, unlike depression in general and postnatal depression, antenatal depression has been infrequently studied with regard to its association with personality traits. Andriola and colleagues [36] found that antenatal depression was significantly related to high HA and low SD, but this study enrolled only 65 pregnant women. The present study aimed to explore the associations between antenatal depression and temperament and character dimensions in a large population of pregnant Japanese women.

## 2. Methods

### 2.1. Participants

All 55 obstetric clinics in Kumamoto Prefecture were solicited to participate in this multiwave study on perinatal mental health. Eighteen (33%) clinics responded to our request. There were one university hospital (the only academia-affiliated hospital in the prefecture), public and private hospitals (*N* = 12), and private clinics (*N* = 5). Hence we obtained a mixture of different types of antenatal institutions. We then invited pregnant women of at least 28 weeks' gestation who attended one of these antenatal clinics during the whole month of November 2011. It is of note that the gestational age in Japan is calculated as week since the first day of the last menstruation. Women were eligible for the study if they were above 20 years old. Pregnant women who were illiterate, who had severe mental illness, or who had been hospitalized with pregnancy complications were excluded. Of 1442 eligible women, 601 (42%) returned the questionnaire while still pregnant.

### 2.2. Measures

#### 2.2.1. Depression

We used the Edinburgh Postnatal Depression Scale (EPDS) [37]. This is a self-report of postnatal depression with 10 items rated on a 4-point scale (from 0 to 3). Its psychometric properties have been reported [37]. The total score ranges from 0 to 30 with higher scores indicating more severe depressive symptoms. The Japanese version of the EPDS was made available by Okano and colleagues [38] who also verified its reliability and validity.

Temperament and character dimensions: we used the 130-item Temperament and Character Inventory [39]. This is based on the original 125-item TCI [10] with the addition of five more PS items in order to increase the internal reliability of the scale, as suggested by Kijima and colleagues [40]. The TCI measures the four temperament dimensions (NS, HA, RD, and PS) and the three character dimensions (SD, CO, and ST). The original true-false response scale was modified into a 4-point scale, which has better internal consistency among Japanese populations [39]; the 4-point scale ranges from 0 (strongly disagree) to 3 (strongly agree). Reliability and factor validity of the Japanese version of the TCI were reported by Kijima and colleagues [40] and Takeuchi and colleagues [41].

#### 2.2.2. Demographic Data

We measured age, partner's age, number of children, complications of pregnancy (such as threatened labour, pregnancy hypertension, placenta previa, and anomaly of the fetus), gestational week, response to the present pregnancy (from very displeased: 1 to very pleased: 5), and desire for baby (from not desired: 1 to very desired: 5).

### 2.3. Statistical Analysis

After examining the means and standard deviations of the variables used in this study, we calculated bivariate correlations between the EPDS scores and the TCI subscale scores and the demographic variables.

In order to identify the relative magnitude of each predictor of depression during pregnancy, we regressed the EPDS scores on predictor variables using the following four steps. First we entered the ages of the participants and their partners. Then we entered the demographic variables that showed significant correlation with the EPDS scores. In the third step, we entered the scores of the character domain scales. This is because we presumed, based on Cloninger and colleagues' [9, 10] assumption, that character domain develops on the basis of temperament domain. Finally, in the fourth step, we entered the scores of the temperament scales.

The criterion for statistical significance was set at *P* < .05. All statistical analyses were conducted using the Statistical Package for Social Science (SPSS) Version 20.

### 2.4. Procedure

All the questionnaires were distributed at the clinics. The participants were asked to bring the questionnaire home, fill it in, and return it to the researcher (T.K.) using a stamp-added envelope. The present study was approved by the Ethical Committee of Kumamoto University Graduate School of Life Sciences.

## 3. Results

The EPDS scores were significantly correlated with NS, HA, ST, and negative response to the current pregnancy. They were significantly negatively correlated with SD, CO, the woman's age, the partner's age, and desire for a baby (Table 1).

In the regression analysis, the EPDS scores were predicted by the woman's and partner's ages, negative response to the current pregnancy, low SD, high ST, high HA, and high PS (Table 2).

Because the Cloninger theory dictates that character develops on the basis of temperament, we entered into a regression analysis the temperament scales before character scales. However, the results were virtually the same (table not shown).

## 4. Discussion

As in Andriola and colleagues' [36] study on postnatal depression as well as other studies on depression in general [6, 7, 13, 15], our study showed that severity of depression during pregnancy was associated with high HA and low SD. Because the effects of the temperament domain on depression remained statistically significant after controlling for the effects of character domains, we could eliminate the possibility that the effects of temperament on depression were wholly explained by mediation of the effects of character domains.

In addition to personality, it was the woman's negative response to the current pregnancy (“I was very displeased”) that significantly predicted the severity of depression during pregnancy. We are not aware of any studies examining the association between such negative psychological attitudes and depression during pregnancy. Much remains to be investigated regarding the temporal sequence of depression during pregnancy and negative psychological reactions towards pregnancy.

Like Cloninger and colleagues' [12] study, our research showed a link between high ST and depression. High ST has reported to be characteristic of individuals with bipolar disorder [13, 42]. Because this study used a self-report of depressive symptoms, we were unable to distinguish individuals with bipolar depression from those with unipolar depression. It is likely that our participants who scored highly on the EPDS included individuals who had previously experienced manic or hypomanic episodes.

As in some studies [12, 13, 18, 19], our results showed a link between high PS and depression during pregnancy. This is, however, in contrast with other studies reporting the reverse association of depression with PS [7, 20–27]. People high in PS are hardworking, persistent, and stable, even in frustrating situations [10]. Coupled with high HA, high PS is associated with obsessive compulsive personality traits [43]. About one-third of people with obsessive compulsive disorder were found to have had current or previous episodes of major depressive disorder [44], and, in about 40% of women with children, obsessive compulsive symptoms had their onset during pregnancy [45]. Depression during pregnancy may be associated with obsessive compulsive traits. Because, however, we failed to measure obsessive compulsive traits or symptoms, our speculation awaits reports in future studies.

Despite the strengths of the present study, including its use of a large community population of pregnant women, its limitations should be discussed. First, caution should be exercised because of the cross-sectional study design. HA is likely to be influenced by the individual's mood at the time of examination [46]. Hence studies with longitudinal follow-up are necessary before firm conclusions can be made regarding the association between personality traits and depression during pregnancy. Alternatively, we could conduct unique statistical analysis applying a nonrecursive model with a “statistical anchor” to fix the effects of an otherwise correlational relationship [47]. Second, the use of a self-report measure of depression made it impossible to distinguish between depressive symptoms and the nosological entity of major depression and allowed for the possible inclusion of individuals with bipolar depression. Another drawback of this study was a low participation rate (42%) though such a high nonparticipation rate was not a rare phenomenon in mental health epidemiologic studies in Japan. For example, in one of the first community studies of mental disorders in Japan [48], the participation rate was 41%. Although it is desirable, we are unaware even of the demographic data such as age and parity of those women who refrained.

Taking these limitations into consideration, the present preliminary study suggests that depression during pregnancy is predicted by high HA, PS, and ST as well as by low SD and women's negative attitudes towards the current pregnancy.

## Figures and Tables

**Table 1:** Means, SDs, and correlations of the variables used in this study (*N* = 608 to 633).

	1	2	3	4	5	6	7	8	9	10	11	12	13	14	15

(1) EPDS	—														
(2) NS	0.15***	—													
(3) HA	0.35***	−0.25***	—												
(4) RD	−0.08	0.3	−0.01	—											
(5) PS	0.08	−0.16***	−0.03	0.11**	—										
(6) SD	−0.56***	−0.23***	−0.48***	0.14**	0.05	—									
(7) CO	−0.29***	−0.22***	−0.25***	0.45***	0.18***	0.50***	—								
(8) ST	0.14**	0.21***	−0.22***	0.01	0.21	−0.11**	0.07	—							
(9) Age	−0.13**	−0.01	−0.11**	−0.07	0.03	0.12**	0.11*	0.07	—						
(10) Partner's age	−0.09*	−0.03	−0.13**	−0.13**	0.03	0.12**	0.05	0.09*	0.68***	—					
(11) No. of children	0.05	−0.04	0.4	−0.03	0.04	−0.02	0.03	−0.03	0.21***	0.14**	—				
(12) Pregnancy complications	0.04	−0.06	−0.01	0.03	0.04	−0.01	0.09*	0.07	0.11**	0.11**	−0.01	—			
(13) Gestational week	−0.02	−0.00	−0.11**	−0.05	0.01	0.03	−0.03	0.04	0.06	−0.00	−0.02	−0.05	—		
(14) Response to pregnancy	0.16***	0.04	0.03	−0.12**	−0.06	−0.11**	−0.09*	−0.08	−0.03	0.01	0.26***	−0.01	0.05	—	
(15) Desire for baby	−0.13**	−0.05	0.00	0.08*	0.02	0.06	0.05	0.01	0.21***	0.15***	−0.18***	0.6	−0.04	−0.47***	—

Mean	4.5	25.5	33.1	29.0	16.3	43.7	50.0	17.2	30.0	32.1	0.7	0.2	34.1	1.3	3.3
SD	4.1	6.0	7.2	4.6	3.0	8.1	6.4	5.8	4.9	5.9	0.8	0.5	3.6	0.6	1.0
Skewness	1.5	−0.1	−0.2	−0.01	0.1	−0.4	−0.1	0.3	0.1	0.7	1.1	2.5	−1.4	2.4	−0.3
Cronbach's alpha									—	—	—	—	—	—	—

Because pregnancy complications and response to pregnancy were more 2.0 positively skewed, both of them were log-transformed for correlations with other variables. *P<0.05, **P < 0.01, and ***P < 0.001.

**Table 2:** Hierarchical multiple regression analyses predicting EPDS score during pregnancy.

	*R* ^ 2^	*R* ^ 2^ increase	Standardised beta	*t*
Step 1: demographics	0.01	0.01*		
Age			−0.07	−1.4
Partner's age			0.03	0.7
Step 2: psychological response	0.04	0.03***		
Response to pregnancy			0.13	3.1**
Desire for baby			−0.02	0.6
Step 3: character	0.33	0.29***		
SD			−0.41	−8.5***
CO			−0.01	0.3
ST			0.11	3.0**
Step 4: temperament	0.37	0.03***		
NS			0.08	1.9
HA			0.19	4.2***
RD			−0.01	−0.3
PS			0.12	3.2**
Standardised *R* ^2^	0.353			

*P < 0.05, **P < 0.01, and ***P < 0.001.

## References

[1] World Health Organization http://www.who.int/mental_health/management/depression/wfmh_paper_depression_wmhd_2012.pdf.

[2] Goldman LS, Nielsen NH, Champion HC (1999). Awareness, diagnosis, and treatment of depression. *Journal of General Internal Medicine*.

[3] Kasen S, Cohen P, Chen H, Castille D (2003). Depression in adult women: age changes and cohort effects. *American Journal of Public Health*.

[4] Akiskal HS, Hirschfeld RMA, Yerevanian BI (1983). The relationship of personality to affective disorders. A critical review. *Archives of General Psychiatry*.

[5] Hirschfeld RMA, Klerman GL, Clayton PJ, Keller MB (1983). Personality and depression. Empirical findings. *Archives of General Psychiatry*.

[6] Jylhä P, Isometsä E (2006). Temperament, character and symptoms of anxiety and depression in the general population. *European Psychiatry*.

[7] Matsudaira T, Kitamura T (2006). Personality traits as risk factors of depression and anxiety among Japanese students. *Journal of Clinical Psychology*.

[8] Klein DN, Kotov R, Bufferd SJ (2011). Personality and depression: explanatory models and review of the evidence. *Annual Review of Clinical Psychology*.

[9] Cloninger CR, Svrakic DM, Przybeck TR (1993). A psychobiological model of temperament and character. *Archives of General Psychiatry*.

[10] Cloninger CR, Przybeck TR, Svrakic DM, Wetzel RD (1994). *The Temperament and Character Inventory (TCI): A Guide to Its Development and Use*.

[11] Celikel FC, Kose S, Cumurcu BE (2009). Cloninger’s temperament and character dimensions of personality in patients with major depressive disorder. *Comprehensive Psychiatry*.

[12] Cloninger CR, Svrakic DM, Przybeck TR (2006). Can personality assessment predict future depression? A twelve-month follow-up of 631 subjects. *Journal of Affective Disorders*.

[13] Farmer RF, Seeley JR (2009). Temperament and character predictors of depressed mood over a 4-year interval. *Depression and Anxiety*.

[14] Furumura K, Koide T, Okada T (2012). Prospective study on the association between harm avoidance and postpartum depressive state in a maternal cohort of Japanese women. *PLoS ONE*.

[15] Nery FG, Hatch JP, Nicoletti MA (2009). Temperament and character traits in major depressive disorder: influence of mood state and recurrence of episodes. *Depression and Anxiety*.

[16] Tanaka E, Kijima N, Kitamura T (1997). Correlations between the temperament and character inventory and the self-rating depression scale among Japanese students. *Psychological Reports*.

[17] Evans L, Akiskal HS, Keck PE (2005). Familiality of temperament in bipolar disorder: support for a genetic spectrum. *Journal of Affective Disorders*.

[18] Farmer A, Mahmood A, Redman K, Harris T, Sadler S, McGuffin P (2003). A sib-pair study of the temperament and character inventory scales in major depression. *Archives of General Psychiatry*.

[19] Hansenne M, Reggers J, Pinto E, Kjiri K, Ajamier A, Ansseau M (1999). Temperament and character inventory (TCI) and depression. *Journal of Psychiatric Research*.

[20] Ghazinour M, Richter J, Eisemann M (2003). Personality related to coping and social support among Iranian refugees in Sweden. *Journal of Nervous and Mental Disease*.

[21] Goncalves DM, Cloninger CR (2010). Validation and normative studies of the Brazilian Portuguese and American versions of the Temperament and Character Inventory—Revised (TCI-R). *Journal of Affective Disorders*.

[22] Jylhä P, Mantere O, Melartin T (2011). Differences in temperament and character dimensions in patients with bipolar I or II or major depressive disorder and general population subjects. *Psychological Medicine*.

[23] Minaya O, Fresán A (2009). Anxiety disorders comorbidity in first-episode depressed patients: personality differences based on the temperament and character inventory. *Personality and Individual Differences*.

[24] Osher Y, Cloninger CR, Belmaker RH (1996). TPQ in euthymic manic-depressive patients. *Journal of Psychiatric Research*.

[25] Osher Y, Lefkifker E, Kotler M (1999). Low persistence in euthymic manic-depressive patients: a replication. *Journal of Affective Disorders*.

[26] Tillman R, Geller B, Craney JL (2003). Temperament and character factors in a prepubertal and early adolescent bipolar disorder phenotype compared to attention deficit hyperactive and normal controls. *Journal of Child and Adolescent Psychopharmacology*.

[27] Josefsson A, Larsson C, Sydsjö G, Nylander P-O (2007). Temperament and character in women with postpartum depression. *Archives of Women’s Mental Health*.

[28] Kitamura T, Shima S, Sugawara M, Toda MA (1996). Clinical and psychosocial correlates of antenatal depression: a review. *Psychotherapy and Psychosomatics*.

[29] Leung BMY, Kaplan BJ (2009). rinatal depression: prevalence, risks, and the nutrition link—a review of the literature. *Journal of the American Dietetic Association*.

[30] Bunevicius R, Kusminskas L, Bunevicius A, Nadisauskiene RJ, Jureniene K, Pop VJM (2009). Psychosocial risk factors for depression during pregnancy. *Acta Obstetricia et Gynecologica Scandinavica*.

[31] Wangel AM, Schei B, Ryding EL (2012). Mental health status in pregnancy among native and non-native Swedish-speaking women: a Bidens study. *Acta Obstetricia et Gynecologica Scandinavica*.

[32] Grigoriadis S, VonderPorten EH, Mamisashvili L (2013). The impact of maternal depression during pregnancy on perinatal outcomes: a systematic review and meta-analysis. *The Journal of Clinical Psychiatry*.

[33] Grote NK, Bridge JA, Gavin AR, Melville JL, Iyengar S, Katon WJ (2010). A meta-analysis of depression during pregnancy and the risk of preterm birth, low birth weight, and intrauterine growth restriction. *Archives of General Psychiatry*.

[34] Byrn MA, Penckofer S (2013). Antenatal depression and gestational diabetes: a review of maternal and fetal outcomes. *Nursing for Women's Health*.

[35] Kitamura T, Yoshida K, Okano T (2006). Multicentre prospective study of perinatal depression in Japan: incidence and correlates of antenatal and postnatal depression. *Archives of Women’s Mental Health*.

[36] Andriola E, di Trani M, Grimaldi A, Donfrancesco R (2011). The relationship between personality and depression in expectant parents. *Depression Research and Treatment*.

[37] Cox JL, Holden JM, Sagovsky R (1987). Detection of postnatal depression. Development of the 10-item Edinburgh Postnatal Depression Scale. *British Journal of Psychiatry*.

[38] Okano T, Murata M, Masuji F (1996). Validation and reliability of Japanese version of EPDS (Edinburgh Postnatal Depression Scale). *Archives of Psychiatric Diagnostics and Clinical Evaluation*.

[39] Kijima N, Saito R, Takeuchi M (1996). Cloninger's seven-factor model of temperament and character and Japanese version of Temperament and Character Inventory (TCI). *Archives of Psychiatric Diagnostics and Clinical Evaluation*.

[40] Kijima N, Tanaka E, Suzuki N, Higuchi H, Kitamura T (2000). Reliability and validity of the Japanese version of the temperament and character inventory. *Psychological Reports*.

[41] Takeuchi M, Miyaoka H, Tomoda A, Suzuki M, Lu X, Kitamura T (2011). Validity and reliability of the Japanese version of the Temperament and Character Inventory: a study of university and college students. *Comprehensive Psychiatry*.

[42] Harley JA, Wells JE, Frampton CMA, Joyce PR (2011). Bipolar disorder and the TCI: higher self-transcendence in bipolar disorder compared to major depression. *Depression Research and Treatment*.

[43] Maggini C, Ampollini P, Marchesi C, Gariboldi S, Cloninger CR (2000). Relationships between tridimensional personality questionnaire dimensions and DSM-III-R personality traits in Italian Adolescents. *Comprehensive Psychiatry*.

[44] Zimmerman M, Coryell W (1989). DSM-III personality disorder diagnoses in a nonpatient sample. Demographic correlates and comorbidity. *Archives of General Psychiatry*.

[45] Neziroglu F, Anemone R, Yaryura-Tobias JA (1992). Onset of obsessive-compulsive disorder in pregnancy. *American Journal of Psychiatry*.

[46] Brown SL, Svrakic DM, Przybeck TR, Cloninger CR (1992). The relationship of personality to mood and anxiety states: a dimensional approach. *Journal of Psychiatric Research*.

[47] Klein R (2005). *Principles and Practice of Structural Equation Modeling*.

[48] Kitamura T, Fujihara S, Iwata N, Tomoda A, Kawakami N, Nakane Y, Radford M (1999). Epidemiology of psychiatric disorders in Japan. *Images in Psychiatry: Japan*.

